# Human Bocavirus in Patients with Encephalitis, Sri Lanka, 2009–2010

**DOI:** 10.3201/eid1911.121548

**Published:** 2013-11

**Authors:** Daisuke Mori, Udaya Ranawaka, Kentaro Yamada, Shaman Rajindrajith, Kazushi Miya, Harsha Kumara Kithsiri Perera, Takashi Matsumoto, Malka Dassanayake, Marcelo Takahiro Mitui, Hisashi Mori, Akira Nishizono, Maria Söderlund-Venermo, Kamruddin Ahmed

**Affiliations:** Oita University, Yufu, Japan (D. Mori, K. Yamada, T. Matsumoto, M.T. Mitui, A. Nishizono, K. Ahmed);; University of Kelaniya, Ragama, Sri Lanka (U. Ranawaka, S. Rajindrajith, H.K.K. Perera);; Colombo North Teaching Hospital, Ragama (M. Dassanayake);; University of Toyama, Toyama, Japan (K. Miya, H. Mori);; University of Helsinki, Helsinki Finland (M. Söderlund-Venermo)

**Keywords:** encephalitis, bocavirus, molecular epidemiology, Sri Lanka, viruses, zoonoses

## Abstract

We identified human bocavirus (HBoV) DNA by PCR in cerebrospinal fluid from adults and children with encephalitis in Sri Lanka. HBoV types 1, 2, and 3 were identified among these cases. Phylogenetic analysis of HBoV1 strain sequences found no subclustering with strains previously identified among encephalitis cases in Bangladesh.

Encephalitis is a serious infection causing high rates of illness and, in industrialized countries, has a case-fatality rate of 6.5%–12% (*1*,*2*). However, the situation in developing countries is largely unknown. Globally, the causes remain unrecognized in 60%–85% of encephalitis cases (*1*,*2*). Recently, human bocavirus (HBoV) has been implicated in causing life-threatening encephalitis in Bangladeshi children (*3*). In Sri Lanka, information about the causative agents of encephalitis is scarce. The aim of this study was to determine the occurrence of HBoV and other possible pathogens in children and adults with encephalitis admitted to a tertiary care hospital in Sri Lanka.

## The Study

The study was conducted at Colombo North Teaching Hospital, Ragama, Sri Lanka, during July 2009–November 2010. A total of 233 patients (110 adolescents/adults >12 years of age and 123 children) were enrolled. Adolescents and adults were admitted to adult wards. Cerebrospinal fluid (CSF) samples were available from 191 patients. Criteria for enrolment were as follows: any combination of the triad of fever, headache, and vomiting, along with altered level of consciousness, seizures, focal neurologic deficits, altered behavior, and signs of meningeal irritation. Clinical and laboratory information was available for 164 patients. The male:female ratio for adolescents/adults was 1.3:1; ages ranged from 12 to 90 years (mean 42 years); For children, the male:female ratio was 0.7:1; ages ranged from 2 to 144 months (mean 48 months). The ethics committees of the University of Kelaniya and Oita University approved this study.

CSF samples were subjected to macroscopic examination, total and differential leukocyte counts, bacterial culture, Gram staining, and measurement of protein and glucose. Blood was cultured for bacteria and examined for total and differential leukocyte counts, erythrocyte sedimentation rates, and hemoglobin and C-reactive protein levels.

Classical encephalitis-causing pathogens (Table) and diarrheagenic viruses, such as HBoV, rotavirus, astrovirus, norovirus, parechovirus, and human adenovirus (HAdV), were determined in CSF by PCR (Technical Appendix) (*3*–*5*). Anti-n-methyl-D-aspartate receptor (NMDAR) encephalitis was diagnosed by on-cell Western analysis (*6*). For HBoV PCR-positive patients, HBoV types 1–4-specific IgG and IgM responses in CSF samples were measured by enzyme immunoassays (*7*).

**Table:** Characteristics of patients with HBoV encephalitis, Sri Lanka, 2009–2010*

Characteristic	Sample no.				
	93018	56684	84770	64502	285
Virus in CSF					
Virus detected†	HBoV1	HBoV1	HBoV1	HBoV2	HBoV3
HBoV IgM and IgG	Neg	Neg	Neg	Neg	Neg
Patient demographic					
Sex	F	F	M	M	F
Age	66 y	46 y	5 mo	17 y	8 mo
Place of residence	Kaleliya	Wattala	Mirigama	Makola	Heiyanthiduwa
Hospitalization					
Time between illness onset and hospitalization	NA	48 h	24 h	48 h	48 h
Duration of hospitalization	7 d	4 d	12 d	4 d	3 d
CSF test result‡					
Color	Clear	Clear	Clear	Clear	Clear
Leukocyte count, cells/μL	1	0	380	0	0
PMNs	0	0	130	0	0
Lymphocytes	1	0	250	0	0
Protein, mg/dL	NA	113	170	38	25
Glucose, mg/dL	65	160	48	63	83
Results of Gram stain	Neg	Neg	ND	Neg	Neg
Bacterial culture	ND	ND	Neg	ND	ND
Blood tests§					
Leukocyte count, cells/μL	10,000	15,200	36,500	15,900	13,200
PMNs, %	63.2	70	62	ND	52
Lymphocytes, %	21.6	21	35	ND	47
Hemoglobin, g/dL	12.2	12	7.7	13.2	13.2
ESR, mm/h	27	68	ND	ND	ND
CRP, mg/dL	ND	ND	>12	ND	<6
Glasgow coma score <15	No	Yes, 12	No	No	No
Outcome	Discharged	Discharged	Discharged	Discharged	Discharged

*HBoV, human bocavirus; CSF, cerebrospinal fluid; Neg, negative; NA, not available; PMN, polymorphonuclear neutrophil: ND, not done; ESR, erythrocyte sedimentation rate; CRP, C-reactive protein.  †The following viruses were tested for herpes simplex virus (HSV) type 1, HSV-2, varicella-zoster virus (HSV-3), Epsetin-Barr virus (human herpesvirus [HHV] type 4), cytomegalovirus (HHV-5), (HHV-6), HHV-7, HHV-8, dengue virus, Japanese encephalitis virus, rubella virus, West Nile virus, yellow fever virus, tick-borne encephalitis virus, Nipah virus, measles virus, mumps virus, parainfluenza virus, respiratory syncytial virus, metapneumovirus, Chikungunya virus, Sindbis virus, Semliki Forest virus, eastern equine encephalitis virus, western equine encephalitis virus, poliovirus, Coxsackie virus, echovirus, enterovirus, lyssaviruses, and Chandipura virus. Bacteria were tested by PCR amplification of 16Sr RNA, followed by sequencing. ‡Reference values: leukocyte count <5 cells/mm^3^ and all lymphocytes; PMNs, none; protein, 20–45 mg/dL; glucose, 50–80 mg/dL or >50% of blood glucose level. §Reference values: leukocyte count, 4,000–11,000 cells/mm^3^; PMNs, 40–60% of leukocyte count; lymphocytes, 20%–40% of leukocyte count; hemoglobin, men: 14–18 g/dL, women, (12–15 g/dL, children: 11–16 g/dL; ESR, <20 mm in1st hr., CRP, <12 mg/dL.

Nucleotide sequences of all amplicons were determined to confirm the PCR products, to distinguish genotypes, and to perform phylogenetic analysis (*3*). BLAST analysis (www.ncbi.nlm.nih.gov/blast) was used to identify the viruses and genotypes. Multiple sequence alignment was conducted by using ClustalW2 (www.ebi.ac.uk/clustalw). The phylogenetic analysis was done with a neighbor-joining tree by using MEGA5 (www.megasoftware.net). A bootstrap analysis of 1,000 replicates was performed to test the reliability of the branching pattern.

The causes of encephalitis were type 2 dengue virus in 1 (0.5%) patient, human echovirus (HEcoV) type 9 or 25 in 2 (1%), HBoV (Table) in 5 (3%), and HAdV 41 in 7 (4%): all were sole detections. None of the other viruses and no bacteria were detected. Samples positive for HBoV by primers designed from viral protein 1/2 also were positive by primers designed from nonstructural protein (NP) 1 gene. HEcoV was detected in 2- and 9-year-old children. HAdV 41 was not confined to children; ages of infected patients ranged from 13 months to 55 years. Of 81 CSF samples, anti-NMDAR encephalitis was detected in 2 (2%) adults (42 and 72 years of age). All patients in this study recovered and were discharged, except for one 13-month-old boy with HAdV 41 encephalitis who left the hospital against medical advice.

The severity of symptoms in the HBoV-positive patients did not differ from those of patients with other infections. None of the patients who had positive PCR results for HBoV1–3 had corresponding HBoV1–4 IgM or IgG in their CSF. Phylogenetic analysis (Figure) of the viral protein 1/2 gene showed that the Sri Lanka HBoV1 strains did not subcluster with encephalitis-associated Bangladesh strain, although they had 97%–98% nt identities. The Sri Lanka HBoV1 strains had 98%–99% nt identities among themselves and with other HBoV1 strains. The Sri Lanka HBoV2 strain was closely related to the Tunisia strain (96% nt identity). The Sri Lanka HBoV2 had 90%–91% nt identities with the Bangladeshi encephalitis-causing strains and 90%–96% nt identities with other HBoV2 strains. The Sri Lanka HBoV3 strain was closely associated with the cluster formed by viruses from the United Kingdom, Australia, Tunisia, and China and had 96%–97% nt identities with those strains. The sequence of NP1 gene is conserved and had 98%–100% nt identities among the Sri Lanka strains.

**Figure:**
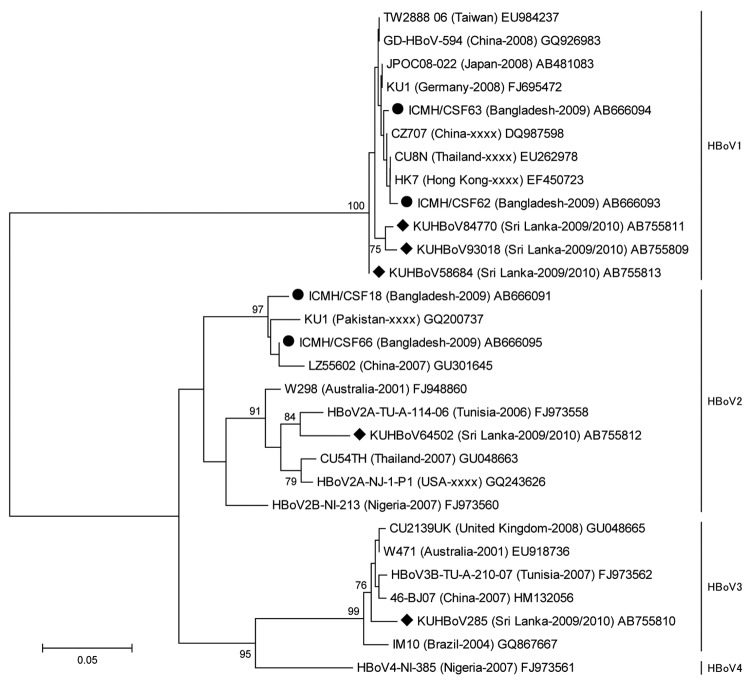
Phylogenetic tree of the partial VP 1/2 gene (nucleotide positions 3233–3808, amplicon size 575 bp) of HBoVs constructed by using nucleotide sequence by neighbor-joining method. The strain name is followed by country of origin and year of sample collection in parentheses, followed by the DDBJ/EMBL/GenBank accession no.; xxx indicates that the year of sample collection is undocumented. Sri Lanka and Bangladesh encephalitis-causing bocaviruses are indicated by circles and diamonds, respectively. The number adjacent to the node represents the bootstrap value, and values <70% are not shown. Scale bar indicates genetic distance expressed as nucleotide substitutions per site. (The DDBJ/EMBL/GenBank accession numbers of nonstructural protein 1 gene of strains KUHBoV93018, KUHBoV285, KUHBoV84770, KUHBoV64502 and KUHBoV58684 were AB822999, AB823000, AB823001, AB823002 and AB823003, respectively).

## Conclusions

The study in Bangladesh suggested that HBoV-associated encephalitis might be restricted to malnourished children (*3*). However, our study demonstrates that HBoV also can be detected in well-nourished children and adults with encephalitis. How HBoV might trigger encephalitis is unclear. HBoV viremia has been documented, and the virus might therefore have the potential to cross the blood–brain barrier. The NP1 of HBoV inhibits interferon-β production by the host, suggesting evasion of the innate immune response during infection (*8*).

Unlike the Bangladesh study, where 2 of 4 encephalitis patients in whom HBoV was detected died (*3*), all patients in our study recovered. In addition to HBoV1 and HBoV2, we detected HBoV3 in a child with encephalitis, which to our knowledge, has not been reported as a cause of the disease. Although HBoV infections occur mainly in children, among the 5 Sri Lanka patients with HBoV encephalitis, 3 were adults or adolescents. None of the patients with HBoV encephalitis had HBoV IgM or IgG in their CSF, indicating how rapidly disease onset occurred and how little time the immune system had to respond. Generally, the specific seroprevalence rate of HBoV1 antibodies in infected persons is 59%, followed by HBoV2, 3, and 4 (34%, 15%, and 2%, respectively) (*7*).

Our detection rate of viruses as a cause of encephalitis was 7.5%, and adding anti-NMDAR encephalitis, the detection rate increased to 10%, which is similar to that of another study (*9*). Anti-NMDAR encephalitis is becoming a dominant cause of encephalitis in certain population (*10*); however, in Sri Lanka, it is 1%–4%, similar to other studies (*11*).

Dengue virus is the leading endemic cause of encephalitis in Brazil (*12*). This infection is also endemic to Sri Lanka and, before our study, dengue encephalitis was suspected but unconfirmed in the population. Enteroviruses frequently cause CNS infection, and the HEcoV 9 and 25 found here are known to cause encephalitis (*13*).

Among the HAdVs, serotype F is mainly responsible for gastroenteritis, whereas encephalitis is caused mainly by serotypes B, C, and D (*14*,*15*). The large number of HAdV 41 encephalitis cases indicates a unique epidemiology in Sri Lanka.

Herpes simplex and varicella-zoster viruses are implicated as the major causes of encephalitis. However, these viruses were not responsible for encephalitis in our study or in the studies in Bangladesh. HBoV is dominant in both Bangladesh and Sri Lanka. The limitation of our study is that causation could not be proven by the presence of HBoV antibody during infection or the absence of HBoV DNA in the CSF when recovered. The HBoV DNA detected in our study may represent persistent DNA from past infection; however, history of recent respiratory or diarrheal infection was absent. Future studies using quantitative PCR and serology are warranted to better establish the etiologic role of HBoV infection and encephalitis.

Technical Appendix Procedures followed to recognize and minimize amplicon contamination during study of human bocavirus in patients with encephalitis, Sri Lanka, 2009–2010.
