# Supplementary material for: Human Bocavirus in Patients with Encephalitis, Sri Lanka, 2009–2010
Source: Emerg Infect Dis. 2013 Nov;19(11):1859–62. doi: 10.3201/eid1911.121548 (PMC3837659; doi:10.3201/eid1911.121548)
Supplement: Technical Appendix — Procedures followed to recognize and minimize amplicon contamination during study of human bocavirus in patients with encephalitis, Sri Lanka, 2009–2010. [file 12-1548-Techapp-s1.pdf]

Article DOI: http://dx.doi.org/10.3201/eid1911.121548

Human Bocavirus in Patients with Encephalitis, Sri Lanka, 2009–2010

Technical Appendix

The following procedures were followed during this study to recognize and minimize amplicon contamination:

1. The nucleic acid extraction, PCR procedures, and running and evaluation of the gel were conducted in separate rooms.

2. A specific set of pipettes, disposable gloves, disposable pipette tips with aerosol filter, racks, and disposable tubes was used for the nucleic acid extraction from the samples or for the PCR procedures, and this set was not used for other purposes. The disposable pipette tips with aerosol filter, the disposable tubes and water used in this study were commercially available and were RNAse and DNAse free. The pipettes, pipette tips, tubes, racks, gloves, and aliquots of water were UV irradiated before and after use. During the procedure, the tubes were kept closed and opened only when necessary. The disposable pipette tips with aerosol filter were used in each step and discarded.

3. The samples were divided into batches, and the nucleic acid was extracted in a biosafety cabinet in a separate room at different intervals to avoid cross contamination.

4. The PCR master mix was prepared and aliquoted, and the templates were added at a clean bench located in a separate room; the template addition was conducted in batches at different intervals to avoid contamination. During the template addition, a negative control without any template was prepared to rule out contamination.

5. Before and after each use, the biosafety cabinet, and the clean bench were decontaminated with alcohol and RNAse Away spray (Molecular Bio-Products, San Diego, CA, USA) to remove RNAse and DNA contamination. The safety cabinet and clean bench were UV irradiated before and after each use.

6. The thermal cycling for the PCR was performed separately in a clean environment. The PCR products were loaded in wells using disposable sterile pipette tips, and a separate tip was used to load each sample. For each batch of samples, a corresponding negative control was run to check for contamination. No contamination was found during our study period.
